# Effect of Antihypertensive Drug Classes on Stroke Prevention: A Systematic Review and Meta-Analysis

**DOI:** 10.7759/cureus.105452

**Published:** 2026-03-18

**Authors:** Zobair Ibn Shams, Kazi Iffat Jahan Maya, Syed Tasin Bin Shahid, Tasnuva Islam Trina, Farha Yasmin Nisat, Fabiha Alam, Azharul Islam, Adiba Afsana, Sayema Chowdhury

**Affiliations:** 1 Department of Acute Medicine, Epsom Hospital, Epsom, GBR; 2 Department of Neuro Medicine, Dhaka Medical College and Hospital, Dhaka, BGD; 3 Department of Geriatric Medicine, Epsom Hospital, Epsom, GBR; 4 Department of Internal Medicine, Dhaka Medical College and Hospital, Dhaka, BGD; 5 Department of Medicine, Wazirpur Upazila Health Complex, Wazirpur, BGD; 6 Department of Cardiology, Liverpool Heart and Chest Hospital, Liverpool, GBR; 7 College of Medicine, University of Wolverhampton, Wolverhampton, GBR; 8 Department of Medicine, Niramoy Poly Clinic, Sylhet, BGD

**Keywords:** antihypertensive agents, comparative effectiveness research, drug therapy, meta-analysis, stroke prevention and control

## Abstract

Hypertension is the most common modifiable risk factor for stroke. However, whether different classes of antihypertensive drugs provide equal protection against stroke remains controversial. This review aimed to systematically compare the effects of various antihypertensive drug classes on stroke prevention through a contemporary meta-analysis of randomized controlled trials (RCTs). Following the Preferred Reporting Items for Systematic Reviews and Meta-Analyses (PRISMA) guidelines, a literature search was conducted in PubMed, Embase, Cochrane Central Register of Controlled Trials (CENTRAL), and Scopus for studies published since January 2000. Eligible RCTs compared angiotensin-converting enzyme inhibitors (ACEIs), angiotensin II receptor blockers (ARBs), beta-blockers (BBs), calcium channel blockers (CCBs), or diuretics with another drug class or placebo and reported stroke incidence. Data were pooled using a random-effects model. Subgroup analyses were performed based on prevention type, baseline risk, and differences in achieved blood pressure. A total of 10 RCTs (N=180,342 participants) were included. The pooled meta-analysis showed no statistically significant difference in stroke risk among the drug classes (risk ratio (RR): 0.94; 95% confidence interval (CI): 0.83-1.05), although heterogeneity was substantial (I²=65%). Subgroup analyses revealed no effect modification by prevention type or baseline risk. However, studies with a moderate to significant between-group difference in systolic blood pressure (SBP) (>2 mmHg) demonstrated a substantial reduction in stroke risk (RR: 0.78; 95% CI: 0.71-0.85), whereas studies with a minimal difference (<2 mmHg) did not (RR: 1.02: 95% CI: 0.90-1.14; p for subgroup difference=0.001). No antihypertensive drug class was found to be superior for stroke prevention overall. The magnitude of achieved SBP reduction, rather than the drug class itself, appears to be the primary determinant of benefit.

## Introduction and background

Stroke is a primary global health concern and remains one of the leading causes of death and long-term disability [1]. Hypertension is the most significant modifiable risk factor for both ischemic and hemorrhagic stroke and shows a substantial, continuous, and independent association with stroke incidence [2]. Pharmacological blood pressure control has played a central role in the primary and secondary prevention of stroke for decades. Numerous clinical trials have consistently demonstrated that antihypertensive therapy reduces blood pressure and, consequently, lowers the risk of first and recurrent stroke [2,3].

Although the benefit of blood pressure reduction is well established, an important and long-standing question in cardiovascular therapeutics is whether all major antihypertensive drug classes provide equal protection against stroke or whether specific pharmacological mechanisms offer additional benefits beyond blood pressure lowering alone [4,5]. The principal drug classes include angiotensin-converting enzyme (ACE) inhibitors, angiotensin II receptor blockers (ARBs), beta-blockers (BBs), calcium channel blockers (CCBs), and thiazide or thiazide-like diuretics. Differences in mechanisms of action may lead to varying effects on central hemodynamics, arterial stiffness, and cerebral autoregulation [4]. Some meta-analyses have suggested that CCBs may offer superior stroke prevention compared with other classes, possibly due to more potent effects on central aortic pressure and pulse wave velocity [2,3,5].

Previous systematic reviews and network meta-analyses have attempted to address this issue; however, the evidence base continues to evolve with the publication of large-scale trials and long-term follow-up data. In addition, comparative effectiveness in specific populations, such as patients with cerebrovascular disease, diabetes, or older adults, requires ongoing synthesis [1-5]. While previous meta-analyses exist, many included older trials with different standards of care, and a contemporary synthesis focusing on modern treatment paradigms is needed. The objective of this study was to provide an updated comparison and to investigate whether apparent class effects are explained by differences in achieved blood pressure. Therefore, an updated and comprehensive systematic review and meta-analysis comparing antihypertensive drug classes for stroke prevention is needed. The present review aims to synthesize high-quality evidence from randomized controlled trials (RCTs) to provide a contemporary comparison of the stroke-preventive efficacy of major antihypertensive drug classes and to inform evidence-based clinical practice and guideline development.

## Review

Methodology

The systematic review and meta-analysis were conducted and reported in accordance with the Preferred Reporting Items for Systematic Reviews and Meta-Analyses (PRISMA) guidelines [6].

*Electronic* *Database* *Search*

A comprehensive and systematic search was performed in four major bibliographic databases: PubMed/MEDLINE, Embase, Cochrane Central Register of Controlled Trials (CENTRAL), and Scopus. The search strategy was structured around four key conceptual components combined using Boolean operators: (1) hypertension, (2) major antihypertensive drug classes, (3) stroke, and (4) a methodological filter to identify RCTs. Controlled vocabulary terms were used where applicable, along with free-text keywords in the title and abstract fields to enhance search sensitivity.

The search was limited to studies published from January 2000 onward to reflect contemporary antihypertensive therapy. The 2000 cut-off was chosen to reflect contemporary clinical practice, including the widespread use of combination therapy and modern blood pressure targets, as established in guidelines from the late 2000s. Only studies involving human participants and published in English were included. Database-specific syntax, field tags, and truncation symbols were adapted for each platform (Table 1).

**Table 1:** Systematic search strategy for electronic databases

Search query components	Applied filters	Syntax/modifiers
#1: Population - Hypertension OR Antihypertensive Agents (MeSH) OR high blood pressure OR arterial hypertension #2: Intervention/Comparison - Angiotensin-Converting Enzyme Inhibitors OR Adrenergic beta-Antagonists OR Calcium Channel Blockers OR Diuretics OR Angiotensin Receptor Antagonists (as MeSH/Emtree terms and keywords) #3: Outcome - Stroke (MeSH) OR Cerebrovascular Disorders OR brain infarction OR cerebral hemorrhage (as keywords) #4: Study Design - Randomized Controlled Trial (pt) OR controlled clinical trial (pt) OR randomized OR placebo OR randomly OR trial OR groups	Final Query: (#1 AND #2 AND #3 AND #4)	Syntax: Boolean operators (AND, OR) were used to combine terms. Modifiers: Truncation () for word variations (e.g., antihypertensiv, random*). Field tags ([MeSH Terms], [Title/Abstract]) were used in PubMed. Publication date filter: From January 1, 2000, to the date of search. Language filter: Restricted to English. Human studies only.

The reference lists of all included trials and relevant previously published systematic reviews were manually screened to ensure that all potentially eligible studies were identified. Two independent reviewers conducted the screening process. Titles and abstracts were first assessed, followed by the full-text evaluation of potentially eligible articles using Rayyan software (Rayyan Systems Inc., Cambridge, Massachusetts, United States) [7]. Disagreements between reviewers at any stage of screening were resolved through discussion and consensus. If consensus could not be reached, a third senior reviewer made the final decision regarding study inclusion or exclusion.

*Application* *of* *the* *PICOS* *Framework* *and* *Exclusion* *Criteria*

The PICOS inclusion criteria were predefined [8]. Only studies involving adult patients with hypertension were included to ensure clinical homogeneity; pediatric studies and trials in which hypertension was not the primary condition were excluded. Regarding interventions, the review focused on head-to-head comparisons among the five major antihypertensive drug classes. Studies comparing agents within the same class or evaluating non-standard treatments as the primary intervention were excluded.

The primary outcome of interest was the incidence of clinical stroke; studies reporting only surrogate markers were excluded. Only long-term RCTs were included to assess sustained preventive effects. Consequently, non-randomized studies, short-term trials, and non-primary research articles were excluded (Table 2).

**Table 2:** Study eligibility and exclusion criteria based on the PICOS framework ACEIs: angiotensin-converting enzyme inhibitors; ARBs: angiotensin II receptor blockers (also known as angiotensin receptor blockers); BBs: beta-blockers (formally, adrenergic beta-antagonists); CCBs: calcium channel blockers; diuretics: (in this context, primarily thiazide and thiazide-like diuretics); TIA: transient ischemic attack; RCTs: randomized controlled trials

PICOS element	Inclusion criteria	Exclusion criteria
Population (P)	Adult participants (≥18 years) with diagnosed primary hypertension (for primary prevention) or a history of cerebrovascular event/TIA (for secondary prevention). Trials enrolling mixed populations (e.g., heart failure, diabetes) were included if data for the hypertensive subgroup or the overall stroke outcome were reported separately.	(1) Studies exclusively in pediatric populations (<18 years). (2) Studies where the population had a non-hypertensive primary indication (e.g., acute myocardial infarction, post-cardiac surgery) unless a distinct hypertensive cohort was analyzed separately. (3) Trials focusing solely on malignant or accelerated hypertension without standard outcome reporting.
Intervention/comparison (I/C)	RCTs directly comparing one major antihypertensive drug class (ACEIs, ARBs, BBs, CCBs, diuretics) against another or against placebo/usual care. Trials comparing combination therapies were included only if the comparison allowed for the isolation of the effect of a specific class (e.g., ACEI + diuretic vs. diuretic).	(1) Trials comparing drugs within the same class (e.g., one ACEI vs. another ACEI) without a different-class comparator. (2) Studies comparing non-standard or non-first-line drug classes (e.g., alpha-blockers, central agonists) as the primary intervention, unless part of a multi-arm trial with relevant comparators. (3) Trials where the comparison involved a non-pharmacological intervention (e.g., diet, exercise) alone without a pharmacological control arm.
Outcome (O)	The primary outcome was fatal or non-fatal stroke incidence, as defined by the original trial protocols. Secondary outcomes included ischemic stroke and hemorrhagic stroke subtypes when reported.	(1) Studies that did not report stroke incidence as a prespecified outcome. (2) Studies reporting only surrogate outcomes (e.g., change in carotid intima-media thickness, blood pressure variability) without clinical stroke events. (3) Case reports, case series, or studies where stroke outcome data were not extractable or provided by the authors upon request.
Study design (S)	Published and unpublished phase III or IV RCTs with a minimum follow-up duration of one year to assess medium- to long-term outcomes. Cluster RCTs and post hoc analyses of RCTs were eligible if they provided original outcome data.	(1) Non-randomized studies (observational cohorts, case-control studies). (2) Reviews, editorials, commentaries, and study protocols without results. (3) RCTs with a follow-up duration of less than one year. (4) Phase I or II trials focusing solely on pharmacokinetics or short-term tolerability.

*Systematic* *Data* *Extraction*

Two reviewers independently extracted data from the included studies using a standardized and piloted electronic data extraction form. The two datasets were compared, and discrepancies were resolved by referring to the original articles and reaching consensus. The following information was collected: (1) study characteristics (first author, year of publication, design, and duration); (2) participant characteristics (sample size, mean age, percentage of females, baseline blood pressure, type of prevention, and comorbidities); (3) intervention and comparator details (specific drug, dosage, and titration schedule); and (4) outcome data (number of stroke events per arm, hazard ratios (HRs) or risk ratios (RRs) with 95% confidence intervals (CIs), and mean achieved blood pressure reduction). When multiple publications reported the same trial, the primary or most comprehensive report was used to avoid data duplication.

*Assessment* *of* *Study* *Quality* *and* *Publication* *Bias*

Two reviewers independently assessed the risk of bias of each included RCT using the revised Cochrane Risk of Bias tool for randomized trials (RoB 2) [9]. Disagreements were resolved through discussion or consultation with a third reviewer. Publication bias for the primary outcome was evaluated by visual inspection of funnel plot asymmetry and further assessed using Egger's linear regression test [10].

*Statistical* *Synthesis* *and* *Analysis*

All statistical analyses were performed using Review Manager (RevMan) version 5.4 (The Cochrane Collaboration, London, England, United Kingdom) [11] and Stata version 17 (StataCorp LLC, College Station, Texas, United States) [12]. Treatment effects for the dichotomous primary outcome (stroke incidence) were expressed as RRs with 95% CIs. For studies reporting time-to-event outcomes, HRs were extracted and pooled. Statistical heterogeneity among studies was assessed using the chi-squared (χ²) test and the I² statistic; I² values greater than 50% indicate substantial heterogeneity.

Primary meta-analyses were conducted using a random-effects model based on the DerSimonian-Laird method, given the expected clinical and methodological diversity across trials. Prespecified subgroup analyses explored potential sources of heterogeneity according to prevention type (primary vs. secondary), baseline vascular risk (high vs. low), and achieved systolic blood pressure (SBP) difference. The analysis based on achieved SBP difference was prespecified, based on the a priori hypothesis that blood pressure (BP) reduction is the main driver of benefit. Sensitivity analyses were performed by excluding studies with a high overall risk of bias.

Results

*Study* *Selection* *Process*

**Figure 1:**
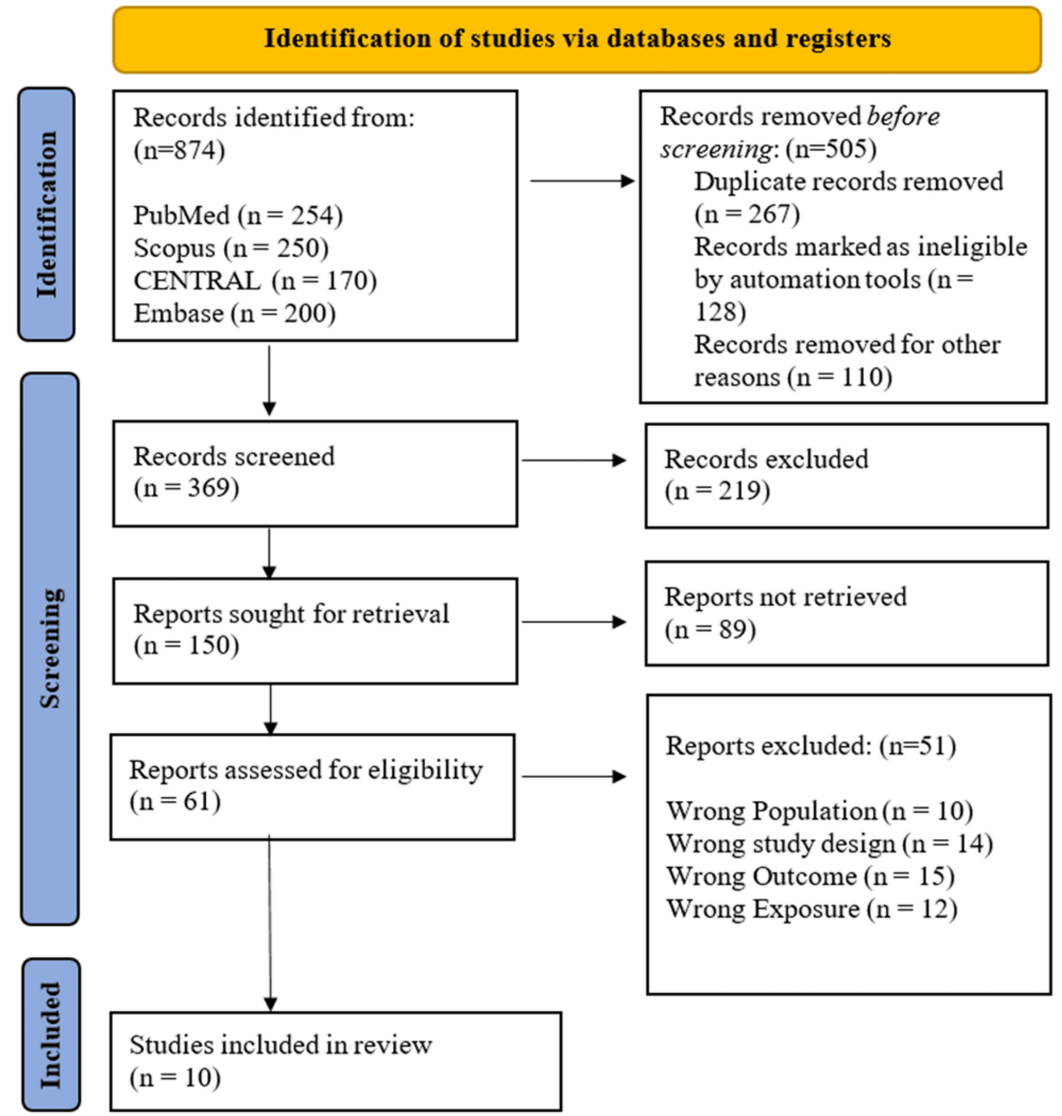
PRISMA flow diagram summary of the systematic literature search and screening PRISMA: Preferred Reporting Items for Systematic Reviews and Meta-Analyses; CENTRAL: Cochrane Central Register of Controlled Trials

A comprehensive search of four electronic databases (PubMed, Scopus, CENTRAL, and Embase) identified 874 records. Before formal screening, 505 records were removed due to being duplicates (n=267), being excluded by automation tools (n=128), and being excluded for other reasons (e.g., language, publication type) (n=110). This left 369 unique records for title and abstract screening, of which 219 were excluded for failing to meet the general eligibility criteria. The remaining 150 records underwent full-text retrieval; however, 89 full-text articles were unavailable. Consequently, 61 full-text articles were assessed for eligibility. Of these, 51 studies were excluded for the following reasons: inappropriate population (n=10), study design (n=14), outcome not reported (n=15), or unsuitable intervention/exposure (n=12). Ultimately, 10 studies met the eligibility criteria and were included in the final systematic review and meta-analysis [13-22] (Figure 1).

The main characteristics of the 10 RCTs included in the meta-analysis are summarized in Table 3. The studies were published between 2000 and 2016 and collectively enrolled over 176,000 participants across primary and secondary prevention settings. Comparisons were made among major antihypertensive drug classes, including diuretics, ACEis, ARBs, CCBs (dihydropyridines and non-dihydropyridines), and BBs, either in head-to-head trials or versus placebo. Follow-up periods ranged from 2.5 to nine years. All studies reported the primary outcome of both fatal and non-fatal stroke, using RRs or HRs as effect measures. Achieved blood pressure data were also reported, allowing quantitative synthesis of the comparative efficacy of the drug classes.

**Table 3:** Characteristics of the included RCTs included in the systematic review and meta-analysis on antihypertensive drug classes and stroke prevention ACEI: angiotensin-converting enzyme inhibitor; ARB: angiotensin II receptor blocker; BB/beta-blocker: beta-adrenergic blocker; BP: blood pressure; CAD: coronary artery disease; CCB: calcium channel blocker; CHD: coronary heart disease; CI: confidence interval; CKD: chronic kidney disease; CV: cardiovascular; CVD: cardiovascular disease; DBP: diastolic blood pressure; HCTZ: hydrochlorothiazide; HR: hazard ratio; LVH: left ventricular hypertrophy; MI: myocardial infarction; NCAS: non-calcium antagonist strategy; Non-DHP CCB: non-dihydropyridine calcium channel blocker (e.g., diltiazem, verapamil); NS: not statistically significant; PROBE: prospective, randomized, open-label, blinded endpoint; RCT: randomized controlled trial; RR: risk ratio or relative risk; SBP: systolic blood pressure; TIA: transient ischemic attack

Author and year	Study design and follow-up		Participant characteristics	Intervention details	Comparator details		Outcome data (stroke)
ALLHAT (2002) [13]	Multicenter, randomized, double-blind, active-controlled trial; mean follow-up ~4.9 years		N=33,357. Mean age ~67; ~47% female. Baseline BP ~145/84 mmHg. Primary prevention. Comorbidities: CHD, diabetes, LVH, etc.	Lisinopril (ACEI) or amlodipine (CCB). Titration to achieve BP <140/90 mmHg. Step 2/3 meds allowed	Chlorthalidone (thiazide diuretic). Same titration goal		Stroke events (fatal + non-fatal): chlorthalidone: 6.3%; lisinopril: 6.3% (RR 1.15, 95% CI 1.02-1.30); amlodipine: 5.6% (RR 0.93, 95% CI 0.82-1.06). Achieved BP: similar across groups
Weber et al. (2010) [14]	RCT; median follow-up ~30 months		N=6,946 with diabetes + hypertension. Mean age: 67.5 yrs. Female: 43%. Baseline BP: ~145/79 mmHg. Comorbidities: diabetes, prior CV events, CKD. Prevention: secondary	Benazepril + amlodipine (B+A). Start: benazepril 20 mg + amlodipine 5 mg. Titration: benazepril → 40 mg; amlodipine → 10 mg if needed	Benazepril + HCTZ (B+H). Start: benazepril 20 mg + HCTZ 12.5 mg. Titration: benazepril → 40 mg; HCTZ → 25 mg if needed		Stroke events: B + A: 64 (1.8%); B + H: 70 (2%). HR: 0.91 (0.65-1.28; p=0.607. Achieved BP: B + A: 131.5/72.6; B + H: 132.7/73.7 mmHg
Lonn et al. (2016) [15]	RCT (2×2 factorial), double-blind, placebo-controlled. Follow-up: median 5.6 years		N=12,705. Mean age: 65.7 years. Female: 46%. Baseline BP: 138.1/81.9 mmHg. Prevention: primary (intermediate risk, no CVD). Comorbidities: intermediate CV risk, low diabetes (5.8%), mild renal dysfunction (2.8%)	Drug: candesartan 16 mg + HCTZ 12.5 mg daily. Titration: fixed dose, no titration	Placebo		Stroke events: active: 75 (1.2%); placebo: 94 (1.5%). HR (stroke): 0.80 (95% CI 0.59-1.08); p=0.14. BP reduction: active vs. placebo: 6.0/3.0 mmHg greater reduction
Skoglund et al. (2015) [16]	Subanalysis of the ACCOMPLISH trial. Follow-up: 36 months		N=11,499. Mean age: 68.4 years. Female: 39.5%. BP at baseline: ~145/80 mmHg. High-risk hypertension	Benazepril + amlodipine (B + A). Dose: benazepril 36 mg, amlodipine 7.7 mg avg.	Benazepril + HCTZ (B+H). Dose: benazepril 36 mg, HCTZ 19.3 mg avg.		Stroke events: B + A: 104 (2.7%); B + H: 66 (1.8%). HR (B + A vs. B + H): 1.22 (0.89-1.78). NS BP reduction: similar in both arms (~1 mmHg difference)
Reisin et al. (2014) [17]	Subgroup analysis of the ALLHAT trial. Follow-up: 4.9 years avg.		N=33,252. Mean age: ~67 years. Female: ~47%. BP at baseline: ~145/83 mmHg. Hypertension + ≥1 CHD risk factor	Chlorthalidone (12.5-25 mg/day)	Amlodipine (5-10 mg/day) or lisinopril (10-40 mg/day)		Stroke events: not stratified by BMI in the paper. Overall stroke HR (amlodipine vs. chlorthalidone): 1.03 (0.93-1.14). NS BP control at 5 years: ~66% in all BMI groups
Ogihara et al. (2015) [18]	PROBE trial (COLM study). Follow-up: 3.3 years median		N=5,141. Mean age: 73.5 years. Female: ~49%. BP at baseline: ~158/87 mmHg. Elderly hypertensive + CV risk	Olmesartan + CCB (amlodipine or azelnidipine)	Olmesartan + diuretic (thiazide)		Stroke events (75-84 y.o.): CCB: 27 (2.4%); diuretic: 42 (3.8%). HR (CCB vs. diuretic): 0.63 (0.39-1.02); p=0.059. BP achieved: ~133/73 mmHg in both arms
Yusuf et al. (2008) [19]	RCT, double-blind, factorial. Follow-up: 2.5 years		N=20,332. Mean age: 66.2 years. Female: ~36%. Baseline BP: ~144/84 mmHg. Prevention: secondary (post-ischemic stroke). Comorbidities: prior stroke/TIA (25%), diabetes (28%), hypertension (74%)	Drug: telmisartan. Dose: 80 mg daily. Titration: fixed dose	Drug: placebo. Dose: matching placebo. Titration: none		Stroke events: 880 (8.7%) vs. 934 (9.2%). Recurrent stroke HR: 0.95 (95% CI 0.86-1.04). BP reduction: -3.8/-2.0 mmHg vs. placebo
Hansson et al. (2000) [20]		PROBE. Follow-up: mean 4.5 years	N=10,881. Mean age: 60.5 years. Female: ~51.5%. Baseline BP: ~173/106 mmHg. Prevention type: primary (hypertensive patients; DBP ≥100 mmHg). Comorbidities: hypertension, some with prior MI, stroke, diabetes (~6.7%)	Drug: diltiazem (non-DHP CCB). Dose: 180-360 mg daily. Titration: stepwise add-on with ACEI, diuretic, or α-blocker if needed	Drug: diuretics and/or β-blockers. Dose: Thiazide or β-blocker first, combined if needed. Titration: stepwise add-on with ACEI or α-blocker; calcium antagonists excluded	Stroke events (diltiazem vs. diuretic/β-blocker): 159 vs. 196 (RR 0.80; 95% CI 0.65-0.99). Mean BP reduction (diltiazem): -20.3/-18.7 mmHg vs. -23.3/-18.7 mmHg (systolic diff: -3.0 mmHg)	
Rothwell et al. (2010) [21]		Post-hoc analysis of two RCTs. Follow-up: median ~5.5 years (ASCOT), up to ~9 years (MRC)	ASCOT: N=19,257. Mean age ~63. MRC: N=4,396. Age 65-74. Female: ~19% (ASCOT). Baseline BP: hypertensive (SBP/DBP not uniformly reported). Prevention: primary prevention of CV events. Comorbidities: Hypertension + ≥3 other vascular risk factors (ASCOT); isolated hypertension in the elderly (MRC)	ASCOT: amlodipine-based (5-10 mg/d), adding perindopril as needed. MRC trial atenolol arm: atenolol (50-100 mg/d), adding HCTZ/nifedipine as needed	ASCOT: atenolol-based (50-100 mg/d), adding bendroflumethiazide/K+ as needed. MRC control: placebo or diuretic (HCTZ + amiloride)	ASCOT stroke events: amlodipine: 279; atenolol: 350. HR for stroke (amlodipine vs. atenolol): 0.78 (0.67-0.90). Adjustment for SBP variability abolished the effect (HR 0.99; 0.85-1.16). Achieved BP reduction: greater SBP reduction with amlodipine; lower BP variability vs. atenolol	
Pepine et al. (2003) [22]		International, randomized, open-label, blinded-endpoint trial. Follow-up: mean 2.7 years	N=22,576. Mean age ~66. Female: ~52%. Baseline BP: ~150/87 mmHg. Prevention: secondary prevention in CAD patients with hypertension. Comorbidities: documented CAD + essential hypertension. High prevalence of diabetes (~30%), prior MI (~32%), and heart failure	CAS: verapamil SR-based (120-480 mg/d). Step 1: verapamil SR. Step 2: add trandolapril (1-8 mg/d). Step 3: titrate doses. Step 4: add HCTZ (12.5-100 mg/d) if needed	NCAS: atenolol-based (25-200 mg/d). Step 1: atenolol. Step 2: add HCTZ. Step 3: titrate doses. Step 4: add trandolapril if needed	Stroke events (non-fatal): CAS: 131; NCAS: 148. RR for stroke (CAS vs. NCAS): 0.88 (0.72-1.07); p=0.33. Primary outcome (death/MI/stroke) RR: 0.98 (0.90-1.06). Achieved BP reduction: similar in both groups. SBP reduction: ~18.8 mmHg. DBP reduction: ~10.1 mmHg	

*Comparative* *Risk*-*of*-*Bias* *Assessment*

**Figure 2:**
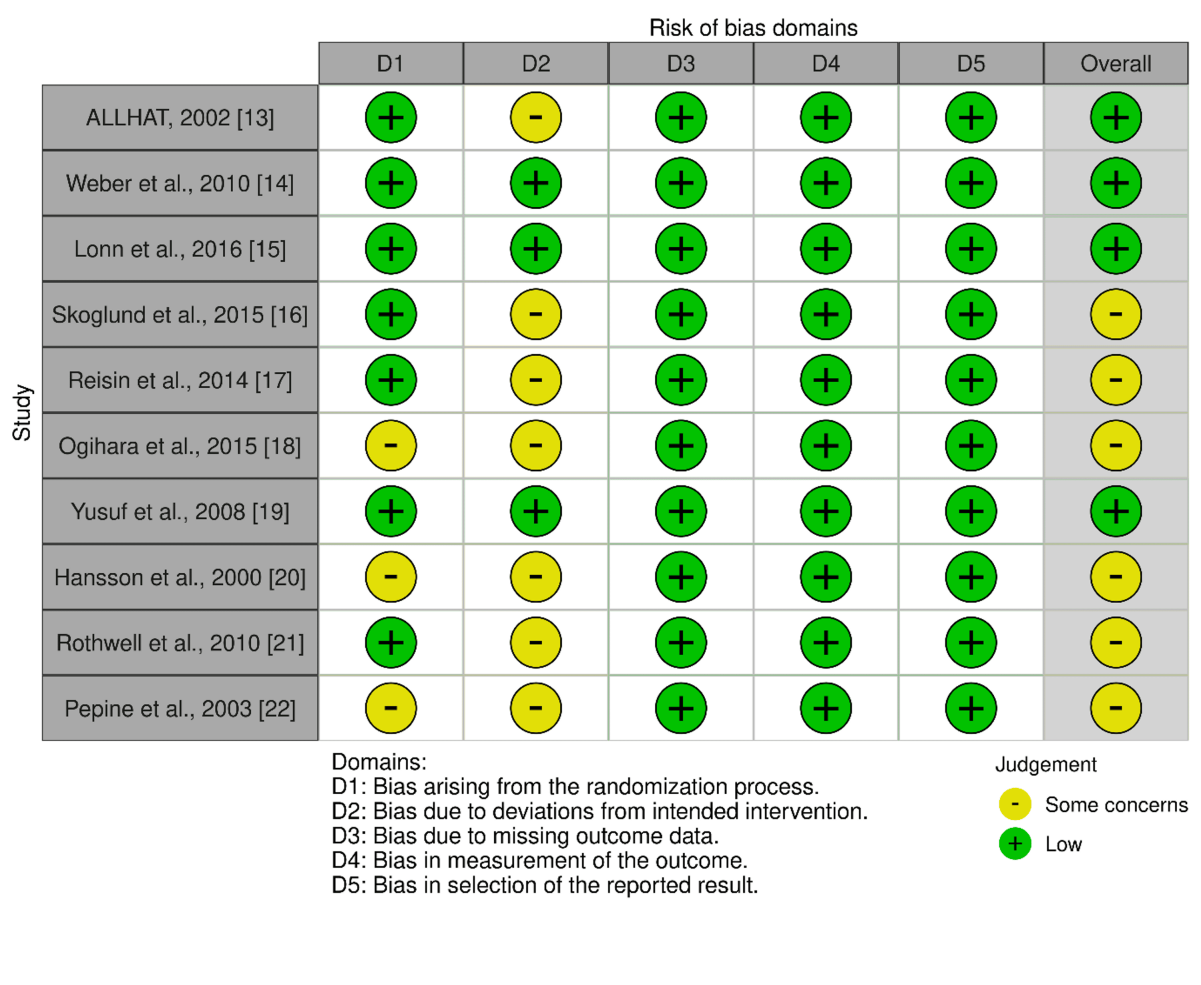
Summary of risk-of-bias assessment for the included studies using the Cochrane RoB 2 tool

Figure 2 presents the risk-of-bias assessment for the 10 included studies. Overall, four studies [13-15,19] were rated as having a low risk of bias. The domain most frequently associated with a high-risk judgment was D2 (deviations from intended interventions), as several open-label or strategy trials were considered at risk for performance bias. All studies were assessed as having a low risk of bias in domains D3, D4, and D5, indicating appropriate handling of missing data, blinded adjudication of outcomes, and reporting of prespecified outcomes.

*Publication* *Bias*

**Figure 3:**
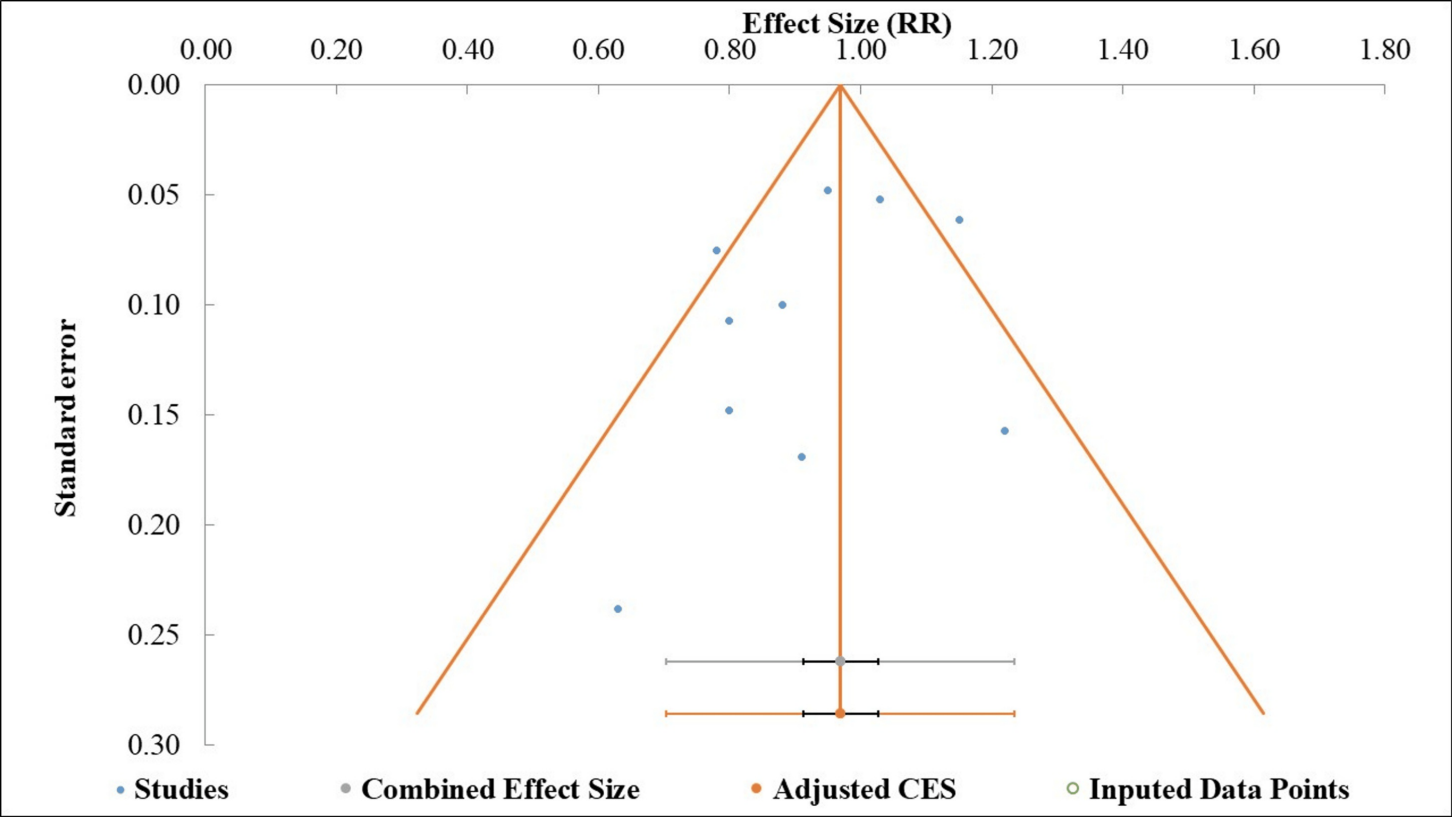
Funnel plot for the assessment of publication bias

Figure 3 presents the funnel plot, which shows a largely symmetrical distribution of study points, suggesting no apparent missing studies. This observation was confirmed by Egger's test (intercept=-1.29; p=0.311), which showed no significant publication bias (Table 4) [10].

**Table 4:** Statistical results of Egger's linear regression test for funnel plot asymmetry

Parameter	Estimate	Std. error	95% CI		t-value	P-value
			Lower limit	Upper limit		
Intercept	-1.29	1.19	-3.99	1.41	-1.08	0.311
Slope	1.06	0.10	0.85	1.28		

*Meta*-*Analysis* *Findings*

**Figure 4:**
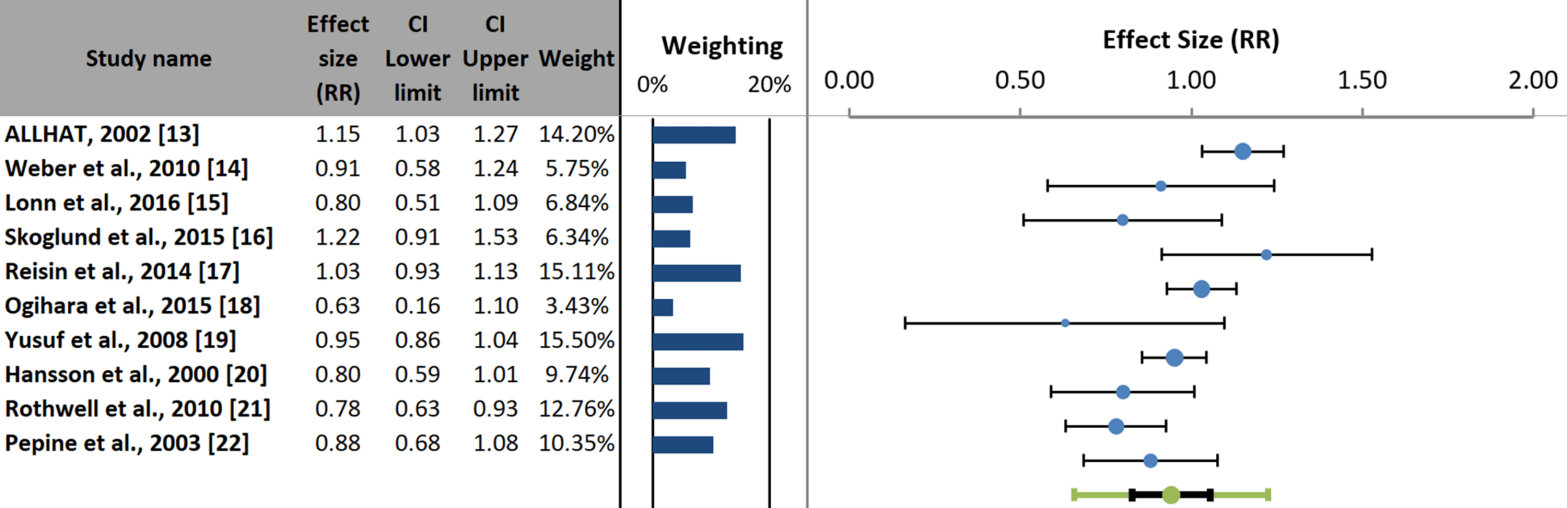
Forest plot of the pooled effect of antihypertensive drug classes on stroke risk

Forest plot and heterogeneity assessment: Figure 4 presents the overall pooled effect of antihypertensive drugs on stroke prevention across all included studies. There was no statistically significant difference between the drug classes, with a pooled RR of 0.94 (95% CI: 0.83-1.05; p>0.05). Substantial heterogeneity was observed among the studies (I²=65.4%; p=0.002) [23].

**Figure 5:**
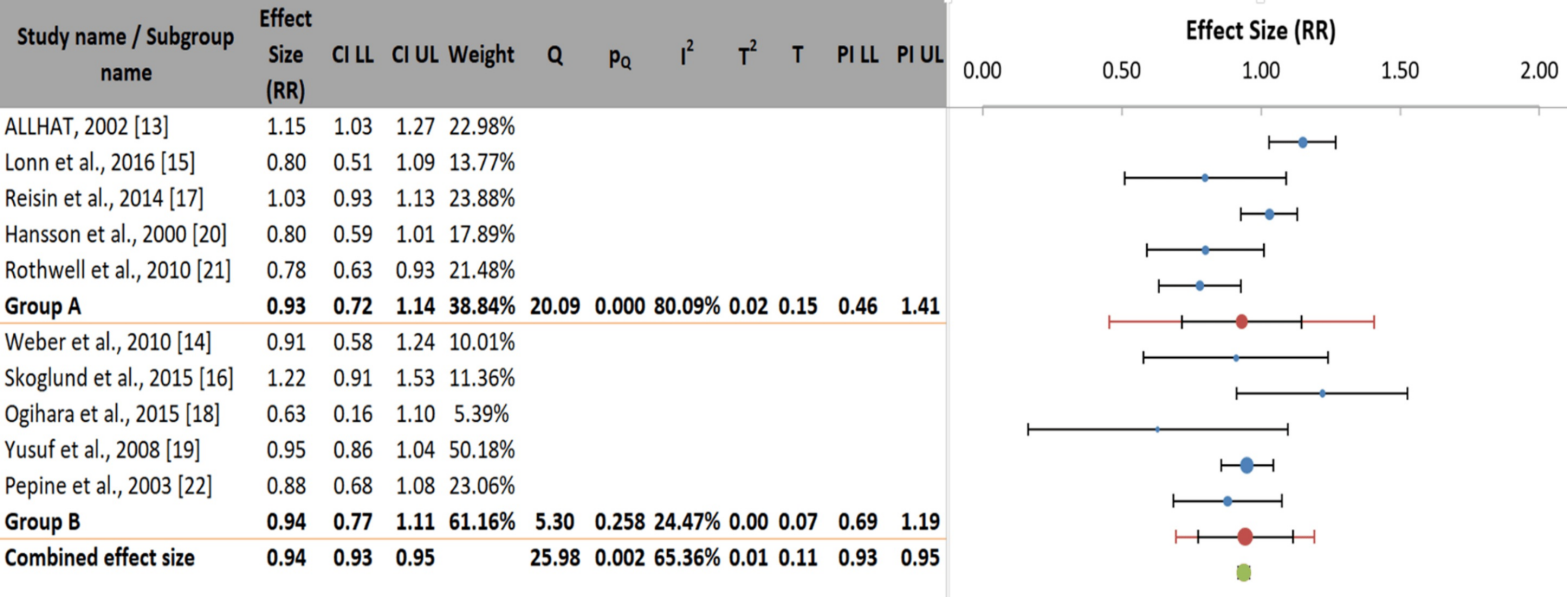
Subgroup analysis by prevention type (primary vs. secondary prevention)

Subgroup analysis: Subgroup analyses showed no statistically significant difference in stroke risk between antihypertensive drug classes for either primary prevention (Group A: RR: 0.93; 95% CI: 0.72-1.14) or secondary prevention (Group B: RR: 0.94; 95% CI: 0.77-1.11). The test for subgroup differences was not significant (p=0.900), indicating that prevention type did not contribute to the overall heterogeneity. Heterogeneity was substantial in the primary prevention subgroup (I²=80.1%; p=0.001), suggesting considerable variation in effect estimates between studies. In contrast, the secondary prevention subgroup exhibited low and non-significant heterogeneity (I²=24.5%; p=0.258) (Figure 5).

**Figure 6:**
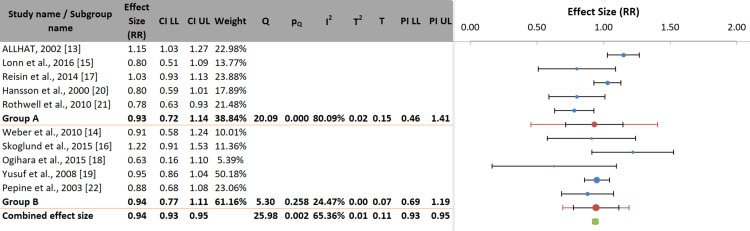
Subgroup analysis by baseline stroke risk (low/moderate vs. very high risk)

Subgroup analysis by baseline stroke risk: The pooled effect estimates showed no statistically significant difference in stroke risk between patients at low to moderately high risk (Group A: RR: 0.93; 95% CI: 0.72-1.14) and those at very high risk (Group B: RR: 0.94; 95% CI: 0.77-1.11). The test for subgroup differences was not significant (p=0.900), indicating that baseline stroke risk did not influence the overall effect of the different antihypertensive drug classes. Heterogeneity was substantial and essential in the low to moderately high-risk subgroup (I²=80.1%; p<0.001), whereas the very-high-risk subgroup showed low and non-significant heterogeneity (I²=24.5%; p=0.258) (Figure 6). This is likely driven by the diversity of populations and comparator arms within this broad category. 

**Figure 7:**
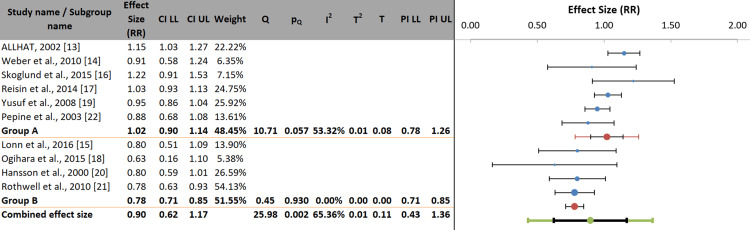
Subgroup analysis by achieved systolic blood pressure difference (minimal vs. moderate to large)

Subgroup analysis by achieved SBP: Figure 7 presents a subgroup analysis of the difference in SBP between the intervention and comparator arms. The test for subgroup differences was statistically significant (p=0.001). Studies with a minimal between-group SBP difference (<2 mmHg) showed no significant reduction in stroke risk (Group A: RR: 1.02; 95% CI: 0.90-1.14). In contrast, studies with a moderate to large SBP difference (2 to >5 mmHg) demonstrated a significant reduction in stroke risk (Group B: RR: 0.78; 95% CI: 0.71-0.85). These findings suggest that the magnitude of BP reduction, rather than the specific drug class, is a key determinant of stroke prevention.

Discussion

This systematic review and meta-analysis synthesized evidence from 10 RCTs involving over 180,000 participants to examine the comparative effects of major antihypertensive drug classes on stroke prevention. The primary finding was that, when pooled, no statistically significant difference existed between drug classes in reducing stroke risk (RR: 0.94; 95% CI: 0.83-1.05). This aligns with longstanding evidence from epidemiological studies, such as the Prospective Studies Collaboration [2], and large-scale meta-analyses, including Law et al. [3], demonstrating that the absolute reduction in BP is the principal mediator of cardiovascular risk reduction, including stroke.

The observed therapeutic equipoise at the class level contrasts with some prior network meta-analyses and observational studies suggesting potential theoretical advantages of CCBs, possibly due to their pronounced effects on central aortic pressure and pulse wave velocity [4]. By incorporating more contemporary head-to-head trials, such as ALLHAT [13] and ACCOMPLISH [14,16], this review indicates that, when effective BP reduction is achieved, no single antihypertensive class demonstrates superiority in stroke prevention across a broad hypertensive population. This supports current guideline recommendations, which emphasize individualized BP management over the preferential selection of any specific drug class [5].

The difference between current study findings and earlier meta-analyses favoring CCBs likely stems from the following: (a) the inclusion of more recent large trials where background therapy and BP targets were more intensive, potentially diluting class-specific effects, and (b) the current study focused on intention-to-treat analysis of drug classes, whereas some earlier work emphasized on-treatment analyses or specific drug combinations.

Despite the overall null effect across drug classes, substantial heterogeneity was observed (I²=65.36%; p=0.002), suggesting that the pooled estimate may not apply equally across all clinical scenarios. This justified the prespecified subgroup analyses to explore potential effect modifiers. Notably, neither prevention type (primary vs. secondary) nor baseline stroke risk significantly influenced treatment effects, indicating that relative efficacy is broadly similar across the spectrum of risk. This observation is clinically reassuring and supports a unified framework for drug selection in both primary and secondary prevention.

The most robust and statistically significant findings emerged in the subgroup analysis stratified by achieved SBP difference. Studies with minimal between-group differences in SBP (<2 mmHg) showed no significant advantage of one drug class over another (RR: 1.02; 95% CI: 0.90-1.14). Conversely, trials achieving a moderate to large SBP reduction (≥2 mmHg) demonstrated a significant 22% relative risk reduction in stroke (RR: 0.78; 95% CI: 0.71-0.85) with a highly significant test of subgroup difference (p=0.001). These results underscore that the magnitude of BP reduction, rather than the pharmacologic class, is the primary determinant of stroke prevention. This aligns with findings by Rothwell et al. [21], which suggested that variations in BP variability across drug classes could confound outcome interpretations when mean BP is comparable.

Subgroup heterogeneity was particularly pronounced in the primary prevention cohort (I²=80.1%), implying that drug-specific characteristics, such as effects on arterial stiffness (favoring CCBs), neurohormonal modulation (favoring renin-angiotensin-aldosterone system (RAAS) inhibitors), or tolerability affecting adherence, may influence outcomes in lower-risk populations, where absolute BP reduction produces more minor effects. In higher-risk, secondary prevention populations, the substantial absolute benefit from BP lowering diminishes the relative impact of these minor drug-specific factors. Additionally, inclusion of strategy trials (e.g., INVEST [22], NORDIL [20]) with protocol-driven add-on therapies reflects real-world treatment escalation but may introduce variability in effect estimates.

Overall, this meta-analysis reinforces that while no mean difference exists between antihypertensive classes in stroke prevention, the extent of BP reduction is the most critical modifiable determinant of benefit. Clinically, this shifts the focus from theoretical superiority of specific drug classes to practical, individualized strategies that reliably achieve target BP goals. Future research should aim to identify patient subgroups who may benefit from particular pharmacologic properties beyond BP lowering.

Limitations

Several limitations merit consideration. First, considerable clinical and methodological heterogeneity existed among the included studies, including variations in specific drugs within the same class, comparator regimens, patient comorbidities, and follow-up duration. These differences were accounted for by using a random-effects model. Second, several key trials employed prospective, randomized, open-label, blinded endpoint (PROBE) designs or compared treatment strategies in which the comparator arm omitted the intervention drug class, potentially introducing performance bias, as noted in the risk-of-bias assessment. Third, the quantitative synthesis included both placebo-controlled and active-comparator trials, some of which involved fixed-dose combinations, which may confound the effect of a single drug class. Third, the subgroup analysis based on achieved SBP difference, while statistically significant and supported by meta-regression, should be interpreted with caution. This analysis relies on study-level aggregate data rather than individual patient data. Consequently, it is subject to the ecological fallacy, in which associations observed at the study level may not accurately reflect the relationship at the individual patient level. Unmeasured confounders, such as baseline SBP, adherence rates, and use of combination therapy, may have influenced both the achieved SBP difference and the observed stroke outcomes. Fourth, the reliance on study-level data rather than individual patient data represents an important limitation. Individual patient data meta-analyses would allow for time-to-event analyses, adjustment for patient-level covariates, and more precise exploration of effect modification by factors such as age, sex, and baseline cardiovascular risk. Our aggregate data approach limits our ability to perform such granular analyses and to definitively establish the causal pathway between drug class, BP reduction, and stroke prevention. Fifth, the included trials rarely reported stroke outcomes separately by pathological subtype (ischemic vs. hemorrhagic). Given that these subtypes have different pathogeneses and may be differentially affected by various antihypertensive mechanisms (e.g., effects on cerebral autoregulation or coagulation), the inability to perform subtype-specific analyses may obscure important class effects. Finally, the restriction to English-language publications and the potential for selective reporting of stroke outcomes in some trials may introduce bias. However, funnel plot symmetry and Egger's test did not suggest significant publication bias for the primary outcome.

Future directions

Future research should prioritize individual patient data meta-analyses to enable time-to-event analyses, adjust for patient-level confounders, and standardize the assessment of achieved BP and visit-to-visit variability. Such analyses should employ causal mediation frameworks to distinguish BP-mediated effects from potential pleiotropic drug class effects formally. Meta-regression should be routinely used to examine continuous moderators, such as differences in SBP, thereby avoiding the limitations of arbitrary subgroup cut-offs. Prospective trial designs must incorporate ambulatory BP monitoring, central aortic pressure, and pulse wave velocity to identify patient phenotypes that may derive differential benefits from specific drug classes independent of BP lowering. Stroke outcomes should be consistently reported by pathological subtype, ischemic versus hemorrhagic, given their distinct pathogeneses. Real-world evidence from international registries, analyzed using target trial emulation and propensity score methods, should complement trial findings by evaluating long-term adherence and effectiveness across diverse, underrepresented populations. Finally, health economic evaluations and patient-centered outcomes, including cognitive function and quality of life, should be integrated into future studies to guide comprehensive clinical decision-making and personalized treatment strategies.

## Conclusions

This contemporary meta-analysis demonstrates that no single antihypertensive drug class is superior to another for stroke prevention when comparing major first-line agents head-to-head. The magnitude of achieved SBP reduction emerged as the critical factor associated with benefit, with trials achieving greater BP differences showing substantially larger stroke risk reductions. These findings suggest that apparent class effects observed in some previous analyses may be largely explained by differential BP-lowering efficacy rather than by unique pharmacological properties. However, these conclusions should be interpreted within the context of substantial heterogeneity across included studies, particularly in primary prevention populations, and the inherent limitations of study-level aggregate data. The inclusion of recent publications from older landmark trials, while necessary for comprehensive analysis, means that some evidence predates contemporary treatment paradigms. Therefore, the choice of antihypertensive therapy should prioritize individualized regimens that effectively achieve and maintain target BP, guided by patient comorbidities, tolerability, and cost, rather than theoretical class-specific advantages for stroke prevention.
